# Predictive and Prognostic Implications of Circulating CX3CR1^+^ CD8^+^ T Cells in Non–Small Cell Lung Cancer Patients Treated with Chemo-Immunotherapy

**DOI:** 10.1158/2767-9764.CRC-22-0383

**Published:** 2023-03-30

**Authors:** Eihab Abdelfatah, Mark D. Long, Ryutaro Kajihara, Takaaki Oba, Takayoshi Yamauchi, Hongbin Chen, Joy Sarkar, Kristopher Attwood, Junko Matsuzaki, Brahm H. Segal, Grace K. Dy, Fumito Ito

**Affiliations:** 1 Department of Surgical Oncology, Roswell Park Comprehensive Cancer Center, Buffalo, New York.; 2 Department of Biostatistics & Bioinformatics, Roswell Park Comprehensive Cancer Center, Buffalo, New York.; 3 Center for Immunotherapy, Roswell Park Comprehensive Cancer Center, Buffalo, New York.; 4 Department of Biomedical Laboratory Sciences, Faculty of Life Sciences, Kumamoto University, Kumamoto, Japan.; 5 Division of Breast and Endocrine Surgery, Department of Surgery, Shinshu University School of Medicine, Matsumoto, Japan.; 6 Department of Surgery, Norris Comprehensive Cancer Center, University of Southern California, Los Angeles, California.; 7 Department of Medical Oncology, Roswell Park Comprehensive Cancer Center, Buffalo, New York.; 8 Department of Medicine, University at Buffalo Jacobs School of Medicine and Biomedical Sciences, The State University of New York, Buffalo, New York.; 9 Department of Immunology, Roswell Park Comprehensive Cancer Center, Buffalo, New York.; 10 Department of Surgery, University at Buffalo Jacobs School of Medicine and Biomedical Sciences, the State University of New York, Buffalo, New York.

## Abstract

Lack of reliable predictive biomarkers is a major limitation of combination therapy with chemotherapy and anti–programmed cell death protein 1/programmed death-ligand 1 (anti-PD-1/PD-L1) therapy (chemo-immunotherapy). We previously observed that the increase of peripheral blood CD8^+^ T cells expressing CX3CR1, a marker of differentiation, correlates with response to anti–PD-1 therapy; however, the predictive and prognostic value of T-cell CX3CR1 expression during chemo-immunotherapy is unknown. Here, we evaluated the utility of circulating CX3CR1^+^CD8^+^ T cells as a predictive correlate of response to chemo-immunotherapy in patients with non–small cell lung cancer (NSCLC). At least 10% increase of the CX3CR1^+^ subset in circulating CD8^+^ T cells from baseline (CX3CR1 score) was associated with response to chemo-immunotherapy as early as 4 weeks with 85.7% overall accuracy of predicting response at 6 weeks. Furthermore, at least 10% increase of the CX3CR1 score correlated with substantially better progression-free (*P* = 0.0051) and overall survival (*P* = 0.0138) on Kaplan–Meier analysis. Combined single-cell RNA/T-cell receptor (TCR) sequencing of circulating T cells from longitudinally obtained blood samples and TCR sequencing of tumor tissue from the same patient who received a long-term benefit from the treatment demonstrated remarkable changes in genomic and transcriptomic signatures of T cells as well as evolution of TCR clonotypes in peripheral blood containing highly frequent tumor-infiltrating lymphocyte repertoires overexpressing *CX3CR1* early after initiation of the treatment despite stable findings of the imaging study. Collectively, these findings highlight the potential utility of T-cell CX3CR1 expression as a dynamic blood-based biomarker during the early course of chemo-immunotherapy and a marker to identify frequent circulating tumor-infiltrating lymphocyte repertoires.

**Significance:**

Current approaches to combined chemotherapy and anti-PD-1/PD-L1 therapy (chemo-immunotherapy) for patients with NSCLC are limited by the lack of reliable predictive biomarkers. This study shows the utility of T-cell differentiation marker, CX3CR1, as an early on-treatment predictor of response and changes in genomic/transcriptomic signatures of circulating tumor-infiltrating lymphocyte repertoires in patients with NSCLC undergoing chemo-immunotherapy.

## Introduction

Immune checkpoint inhibitor (ICI) therapy has become a major component in the management of non–small cell lung cancer (NSCLC) in recent years. Inhibitory immune checkpoints, such as the programmed cell death 1 (PD-1)/programmed death-ligand 1 (PD-L1) pathway, function to prevent healthy tissue from excessive immune-mediated destruction during inflammatory states. By hijacking this machinery, tumor cells can evade detection and elimination by the immune system. ICIs that block PD-1/PD-L1 axis have demonstrated improvements in survival over chemotherapy alone in NSCLC in multiple randomized trials both when administered as individual agents (1–3) and as combination ICI regimens (4). This has led to FDA approval of pembrolizumab, nivolumab, and atezolizumab in the treatment of NSCLC, and approval of single-agent pembrolizumab as first-line therapy for PD-L1–positive advanced NSCLC (5). In addition, combination chemo-immunotherapy regimens have shown improved outcomes over chemotherapy alone in several recent clinical trials (6–8).

High PD-L1 expression in the tumor microenvironment (TME) has been correlated with response to PD-1/PD-L1 blockade therapy and is used as a biomarker for the initiation of immunotherapy in NSCLC (1, 4, 9). However, no established biomarker presently exists for chemo-immunotherapy. In trials that showed improvements in survival with combination chemo-immune regimes, PD-L1 expression was not found to be correlated with response to treatment, and there were significant improvements in survival even in patients with PD-L1 expression < 1% (6, 8). Other criticisms of PD-L1 expression include variability in different testing platforms and the intratumoral heterogeneity of PD-L1 expression (10). Other biomarkers for immunotherapy, such as tumor mutational burden and tumor-infiltrating lymphocytes (TIL), have been proposed, although these are still under investigation with varying results (10).

Despite the improved responses seen with chemotherapy and anti–PD-1/PD-L1 therapy (chemo-immunotherapy), only a fraction of patients show durable response with improved survival even with high PD-L1 expression (6–8). In addition, there are serious immune-related toxicities associated with ICI therapy due to nonspecific activation of the immune system by ICIs. These toxicities can affect any organ system in the body and are sometimes irreversible, and most commonly include pneumonitis, colitis, hepatitis, dermatitis, hypothyroidism, and hypophysitis (11). In combination chemo-immune regimens, there are chemotherapy-related side effects in addition to those of ICIs. Therefore, establishing an effective biomarker to predict response to chemo-immunotherapy is important not only to optimize treatment, but also avoid or minimize serious toxicity.

CX3C chemokine receptor 1 (CX3CR1) is widely expressed in immune cells including dendritic cells, monocytes, macrophages, natural killer (NK) cells, and T cells (12). Expression of CX3CR1 correlates with the degree of effector CD8^+^ T-cell differentiation (13). Accumulating evidence has revealed the unique features of CX3CR1 suitable for use as a blood-based T-cell biomarker for immunotherapy. First, despite higher expression of cytotoxic effector molecules, granzymes and perforin, and potent cytotoxicity *in vitro*, CX3CR1^+^CD8^+^ T cells show lower expression of CXCR3 and L-selectin (CD62L; refs. 13–17), trafficking receptors needed for entry across the tumor microvasculature and lymphoid organ high endothelial venules, respectively (18–20), which allows the CX3CR1^+^ subset to remain in circulation after initial response while CX3CR1^–^ subsets traffic to the TME and mediate antitumor efficacy (13, 15). Second, unidirectional differentiation of CX3CR1^–^ CD8^+^ T cells to CX3CR1^+^ CD8^+^ T cells (13–15) facilitates stable expression of CX3CR1 in CD8^+^ T cells in the effector phase unlike other molecules transiently upregulated after activation such as PD-1, 4-1BB, ICOS, and Ki67. Consequently, our study and others have demonstrated that tumor-specific and tumor-infiltrating CD8^+^ T-cell repertoires are enriched in the circulating CX3CR1^+^ CD8^+^ subset in preclinical models and patients (16, 21), and the frequency of peripheral blood (PB) CX3CR1^+^ CD8^+^ T cells increases after effective immunotherapy such as adoptive T-cell therapy, neoantigen/*in situ* vaccination, and ICI therapy (15, 17, 22, 23).

We have recently analyzed longitudinal PB samples from patients with NSCLC undergoing anti–PD-1 therapy, and found that the percent change of the CX3CR1^+^ subset in PB CD8^+^ T cells from baseline (CX3CR1 score) associated with response and prognosis (16). Although it remains unclear whether T-cell CX3CR1 is a predictor of response to combination chemo-immunotherapy, Yan and colleagues demonstrated that CX3CR1^+^CD8^+^ T cells withstand treatment with chemotherapy and are increased in response to chemo-immunotherapy in patients with metastatic melanoma (22). These findings set the stage for us to explore the utility of PB CX3CR1^+^CD8^+^ T cells as a potential biomarker for treatment response to chemo-immunotherapy in patients with NSCLC.

Here, we hypothesize that the change of the CX3CR1^+^ subset in PB CD8^+^ T cells from baseline correlates with response to chemo-immunotherapy in patients with advanced NSCLC. We collected serial PB samples from patients with advanced NSCLC undergoing treatment with chemo-immunotherapy, analyzed levels of the CX3CR1^+^ subset in CD8^+^ T cells, and evaluated its predictive and prognostic values of the CX3CR1 score. Furthermore, we employed T-cell receptor sequencing (TCR-seq) of pretreatment tumor tissue and single-cell (sc) RNA/TCR-seq of serial PB samples to evaluate genomic and transcriptomic signatures of tumor-infiltrating lymphocyte repertoires in PB. Our studies identify a potential role of T-cell CX3CR1 as a predictor of response to chemo-immunotherapy in patients with NSCLC.

## Methods

### Study Design, Patients, and Specimen Collection

Informed written consent was obtained from 29 patients with naïve or previously treated NSCLC, undergoing combination of chemotherapy and anti-PD-1/PD-L1 antibody (pembrolizumab or atezolizumab) for the collection and storage of blood samples, the analysis of archived tumor tissue, and the review of their medical records under the Institutional Review Board of Roswell Park Comprehensive Cancer Center protocol approval (I 188310), in accordance with the Declaration of Helsinki.

### Data Reporting

The clinical samples were prospectively collected, and selected on the basis of availability during the study window. No statistical methods were used to predetermine sample size. Randomization was neither feasible nor appropriate due to the nature of this study. The observed sample size (*n* = 29) provides an adequate pool of both responders and non-responders, and produces performance measures (i.e., sensitivity, ROC curves, and the corresponding AUC, etc.) with adequate levels of precision. We evaluated the correlation between a biomarker performance during treatment and response. The investigators were not blinded during experiments and outcome assessment. This study was conducted in accordance with the Reporting Recommendations for Tumor Marker Prognostic Studies (REMARK; ref. 24).

### Flow Cytometry

PB was obtained in EDTA-containing tubes. Peripheral blood mononuclear cells (PBMCs) were isolated using Lymphocyte Separation Medium (Corning) density gradient centrifugation. Fresh or cryopreserved PBMCs were incubated with anti-human IgG (Sigma). These antibodies were used for flow cytometry or cell sorting: anti-human CD3 (clone UCHT1; BioLegend), CD4 (clone RPA-T4; BD Biosciences), CD8 (clone RPA-T8; BioLegend), CD45 (clone HI30; BD Biosciences), CD56 (cone HCD56: BioLegend), CD19 (clone HIB19 BioLegend), and CX3CR1 (clone 2A9–1; BioLegend) antibodies. Samples were acquired using LSRFortessa (BD Biosciences), and data were analyzed with FlowJo software v10.1.5 (FlowJo LLC).

### Assessment of Response

Clinical response to chemo-immunotherapy was assessed as best response according to immune-related RECIST (iRECIST; ref. 25) within 12 weeks as described previously (16). Patients who had complete response (CR) and partial response (PR) were classified as responders while ones with stable disease (SD) and progressive disease (PD) as nonresponders. Objective responses were confirmed by at least one sequential tumor assessment. Overall response rates (ORR) were calculated as [(CR + PR) ÷ number of patients] × 100.

### IHC Studies

The expression of PD-L1 on the surface of tumor cells was reported as a standard of care before treatment, which was performed using the 22C3 PharmDx antibody on the Dako Omnis platform (Agilent) and scored by published guidelines (26).

### scRNA/TCR-seq

#### Sample Preparation and scRNA/TCR-seq Library Generation

Single live CD45^+^CD19^–^CD3^+^CD56^–^ cells were enriched from cryopreserved PBMC samples by flow cytometry sorting using a BD FACSAria II (BD Biosciences; Supplementary Fig. S1A and S1B). Cells were counted, hashed with TotalSeq-C0251 (ref: 394661), TotalSeq-C0252 (ref: 394663), TotalSeq-C0253 (ref: 394665), and TotalSeq-C0254 (ref: 394667) anti-human antibodies (BioLegend) and pooled. The pooled cells were then quantified and subsequently loaded onto the chromium chip G using the standard protocol for the Chromium single-cell 5′ kit v2 (10x Genomics, Inc). Following Gel Bead-in Emulsion (GEM) generation, samples were processed according to the standard manufacturer's protocol. After sequencing libraries passed standard quality control metrics, the libraries were sequenced on Illumina NovaSeq6000 S1 100cycle v1.5 kits with the following read structure: read1: 28, read2: 90, index 1: 10, index 2:10. Libraries were sequenced to obtain a read depth greater than 16,000 reads/cell for the gene-expression (GEX) libraries and greater than 4,000 reads/cell for the V(D)J-enriched T-cell libraries.

#### Raw Data Processing, Quality Control, and Subsequent Analyses

Raw sequence data demultiplexing, barcode processing, alignment (GRCh38), and filtering for true cells were performed using the Cell Ranger Single-Cell Software Suite (v6.0.0), yielding 8,916 cells (pretreatment: 4,170 cells, 3 weeks: 534 cells, 6 weeks: 3,090 cells, 9 weeks: 1,122 cells) with a mean of 22,239 reads/cell (90.15% mapping rate), median of 1,175 genes/cell, 19,831 total unique detectable genes, and 2,697 median UMI counts/cell. Seurat (v4; ref. 27) was used to perform filtering, normalization, and downstream analyses as previously described (refs. 23, 28; Supplementary Fig. S1C–S1F). Hashtag feature barcoding (TotalSeq-C antibodies, BioLegend) of pooled samples was demultiplexed using a k-medoid clustering approach implemented by Seurat to assign cells to individual samples and remove doublets. After quality control assessment, 4,040 cells were removed (45.32% of total cells), and 4,876 high quality cells (pretreatment: 1,861 cells, 3 weeks: 223 cells, 6 weeks: 2,137 cells, 9 weeks: 655 cells) were included in downstream analyses. VDJ annotations derived from Cell Ranger were analyzed using scRepertoire (29) and custom scripts. Differential TCRB clonotype abundances were determined by Fisher exact test. Gene-set enrichment analysis (GSEA) of cluster-specific gene markers was performed via enrichR (30). Reference gene sets included those from the GO-Biological Processes and Reactome databases, compiled from the Molecular Signatures Database (MSigDB; ref. 31). Gene sets with Benjamini–Hochberg adjusted *P* < 0.05 were considered as significantly enriched.

### TCR Sequencing

We obtained DNA from NSCLC formalin-fixed, paraffin-embedded (FFPE) samples prepared within the 2 months before the initiation of chemo-immunotherapy. TCRβ CDR3 repertoires were profiled using the ImmunoSEQ immune profiling platform at the survey level (Adaptive Biotechnologies) as previously described (16, 23). Repertoire characteristics were analyzed using the LymphoSeq package and custom scripts in the R statistical software environment. The level of similarity (Morisita–Horn Index) between repertoires was calculated using the vegan package.

### Statistical Analysis

Patient demographic and clinical characteristics were reported using the mean and range for continuous variables; and frequencies and relative frequencies for categorical variables. The marker expression was compared using the Mann–Whitney *U* test as described before (16). The maximal percent change of the CX3CR1^+^ subset in PB CD8^+^ T cells from baseline by the given time point was calculated as described previously (16). We assessed the correlation between the maximal percent change in CX3CR1 and objective response as described previously (16). Briefly, we estimated the ROC curves and the corresponding AUC using a logistic regression model, and obtained confidence intervals for the AUC using DeLong method (32). We utilized the Youden index criterion (33) to identify the optimal cut-off point, and examined sensitivity, specificity, positive predictive value (PPV), and negative predictive value (NPV); with 95% confidence intervals by Jeffrey prior method. Survival outcomes were summarized by groups (i.e., CX3CR1 expression) using standard Kaplan–Meier methods, with comparisons made using the log-rank test (GraphPad Prism 9.4.1). Associations with demographic and clinical factors were assessed using Cox regression models, where HRs were obtained from model estimates. The variables that were found to be *P* < 0.01 on univariate analysis were included in the multivariable analysis. Fisher exact test was used to assess the association between PD-L1 expression or the CX3CR1 score and objective response.

### Data Availability

Raw and processed scRNA/TCR-seq data supporting the findings of this study have been deposited in the National Center for Biotechnology Information Gene Expression Omnibus (NCBI-GEO) under accession number GSE213902. Bulk TIL TCRseq clonotype calls derived from Adaptive ImmunoSEQ are available at https://github.com/mdlong-rpccc/Ito_ChemoImmunotherapy_CX3CR1. Other source data are provided with this article. All data generated and analyzed are available from the corresponding author upon reasonable request.

## Results

### Expansion of PB CX3CR1^+^CD8^+^ T Cells Early After Initiation of Chemo-immunotherapy Correlates with Response and Better Prognosis in Patients with NSCLC

We evaluated 29 patients who were treated with chemo-immunotherapy, and enrolled in this study between February of 2018 and July of 2021. The cut-off date for treatment outcome analysis was October 30, 2022, at which time response evaluations were available for 29 patients. The median time of follow-up was 35.7 months (range 3.3–43.4). Baseline characteristics of 29 patients are shown in Supplementary Table S1. The majority of patients (17 patients; 59%) received carboplatin, pemetrexed and pembrolizumab while 10 patients (34%) received carboplatin, paclitaxel and pembrolizumab, and 2 patients (7%) received carboplatin, paclitaxel, atezolizumab, and bevacizumab. Twelve patients (41%) were classified as “responders” due to partial response (PR) as their best response within 12 weeks from the initiation of therapy by iRECIST criteria while no patients achieved a complete response (CR). Seventeen patients (59%) were classified as “nonresponders”, and of these 13 had stable disease (SD) while 4 demonstrated progressive disease (PD) based on iRECIST criteria, resulting in an ORR of 41.3%.

**FIGURE 1:**
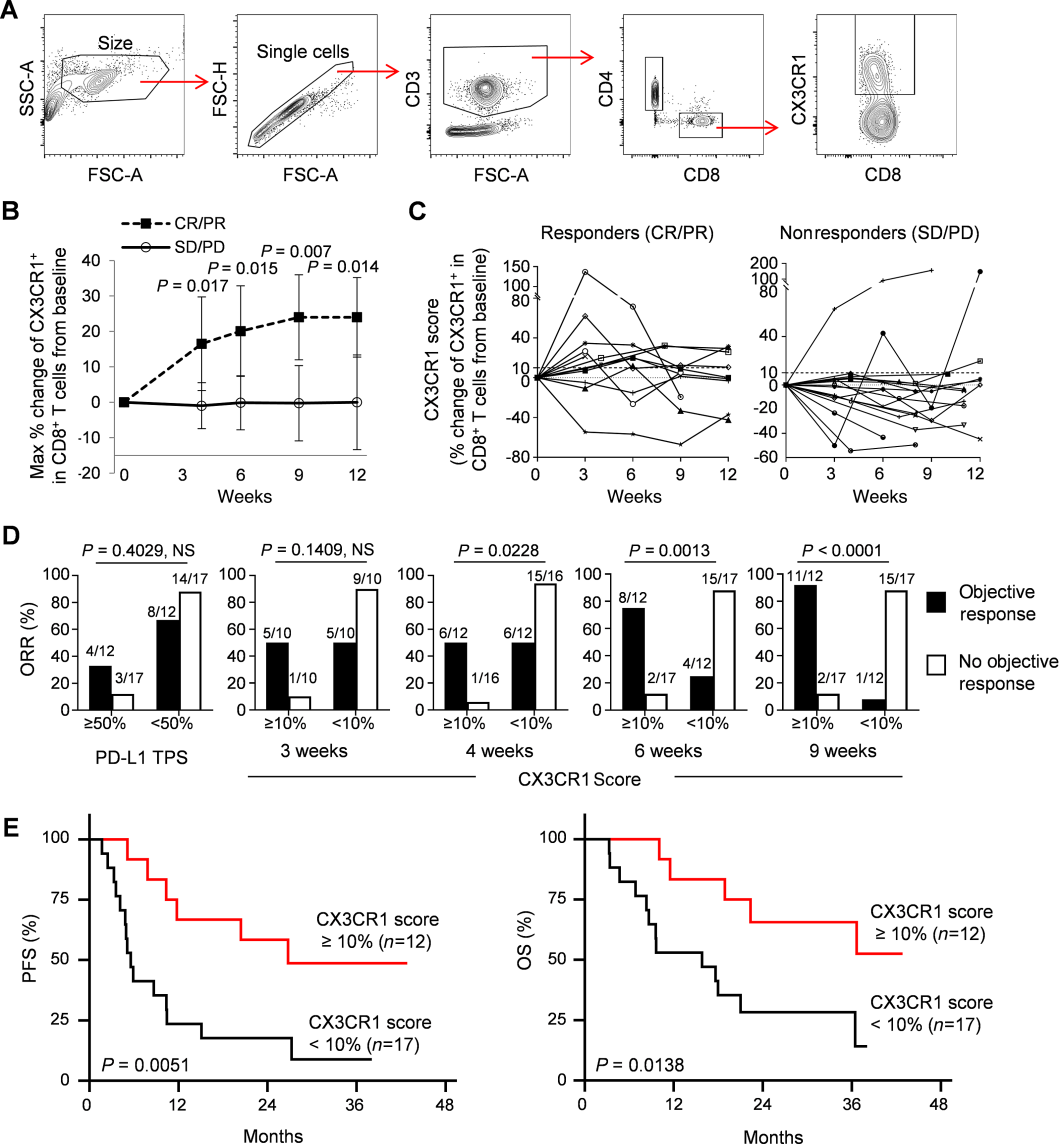
Expansion of PB CX3CR1^+^CD8^+^ T cells early after initiation of chemo-immunotherapy correlates with response and better prognosis in patients with NSCLC. **A,** Gating strategy for identifying CX3CR1^+^ CD8^+^ T cells in PBMCs. **B,** The largest percent change of the CX3CR1^+^ subset in PB CD8^+^ T cells from baseline by the given time point in responders (CR/PR: *n* = 12) and nonresponders (SD/PD: *n* = 17) of 29 patients with NSCLC treated with chemo-immunotherapy. *P* values were calculated by a two-tailed Mann–Whitney *U* test. Values are median ± SEM. **C,** Percent change of the CX3CR1^+^ subset in PB CD8^+^ T cells from baseline (CX3CR1 score) in responders and nonresponders. **D,** Overall response rate (ORR) for high and low PD-L1 tumor proportion score (TPS) and PB CX3CR1 score at 3, 4, 6, and 9 weeks. ORR was analyzed by Fisher exact test. NS, not significant. **E,** PFS and OS for high (≥10%) versus low (10%) CX3CR1 score. *P* values were calculated by a log-rank (Mantel–Cox) test.

Next, we analyzed the frequency of the CX3CR1^+^ subset in PB CD8^+^ T cells (Fig. 1A). The median baseline frequency of the CX3CR1^+^ subset among CD8^+^ T cells was 30.9% (4.0–85.1%) with no difference in ORR and progression-free (PFS) and overall survival (OS) between the low- and high-frequency groups using various cut-off points (Supplementary Fig. S2). As described recently, we analyzed responses in terms of the percent change of the CX3CR1^+^ subset in circulating CD8^+^ T cells from baseline (CX3CR1 score; ref. 16). The maximal percent change of the CX3CR1^+^ subset in PB CD8^+^ T cells from baseline by the given time point could differentiate responders from nonresponders as early as 4 weeks from the start of the treatment (Fig. 1B). Next, an ROC curve and the Youden Index (33) were used to determine the optimal cut-off score of the maximal percent changes in CX3CR1 biomarker expression on PB CD8^+^ T cells. We found that an increase of CX3CR1^+^ CD8^+^ T-cell subsets by 9.42%–10.35% from baseline differentiated responders from nonresponders at 6–12 weeks, and was correlated with higher OR, sensitivity, specificity, PPV, and NPV (Supplementary Table S2). In line with this finding, at least 10% increase of the CX3CR1 score was observed in 83.3% (10/12) of responders compared with 10.5% (2/19) of nonresponders (Fig. 1C). Indeed, at least 10% increase of the CX3CR1 score started to correlate with ORR at 4 weeks [*P* = 0.0228; OR, 10.7; 95% confidence interval (CI), 1.05–109.78], and became more predictive at 9 weeks (*P* < 0.0001; OR, 15; 95% CI, 2.24–100.48; Fig. 1D; Supplementary Table S3). We then analyzed the sensitivity, specificity, PPV, NPV, and accuracy of our biomarker at various time points and compared them to PD-L1 expression by tumor proportion score (TPS). The maximal CX3CR1 score of at least 10% increase exhibited higher sensitivity, specificity, PPV, and NPV than the PD-L1 TPS, and identified response in 14/20 (70.0%), 22/27 (81.4%), 24/28 (85.7%) and 25/29 (86.2%) at 3, 4, 6, and 9 weeks, respectively (Table 1).

**TABLE 1:** Comparison of biomarker performance between PD-L1 TPS and the CX3CR1 score

		PB CX3CR1 score ≥10%			
	PD-L1 TPS ≥50% (*n* = 25)	At 3 weeks (*n* = 20)	At 4 weeks (*n* = 28)	At 6 weeks (*n* = 29)	At 9 weeks (*n* = 29)
PPV	57.1% (4/7)	83.3% (5/6)	87.5% (7/8)	83.3% (10/12)	83.3% (10/12)
NPV	63.6% (14/22)	64.3% (9/14)	75.0% (15/20)	87.5% (14/16)	88.2% (15/17)
Sensitivity	33.3% (4/12)	50.0% (5/10)	58.3% (7/12)	83.3% (10/12)	83.3% (10/12)
Specificity	82.4% (14/17)	90.0% (9/10)	93.8% (15/16)	87.5% (14/16)	88.2% (15/17)
Accuracy	62.1% (18/29)	70.0% (14/20)	81.4% (22/27)	85.7% (24/28)	86.2% (25/29)

Abbreviation: CX3CR1 score, percent change of the CX3CR1^+^ subset in peripheral blood CD8^+^ T cells from baseline.

Next, we evaluated relevant variables for correlation with PFS and OS. Kaplan–Meier survival analysis demonstrated significantly improved PFS (*P* = 0.0051) and OS (*P* = 0.0138) by log-rank test for patients with at least 10% increase in CX3CR1 score (Fig. 1E). Of note, we previously reported that 20% increase in CX3CR1 score correlated with survival in a patient with NSCLC undergoing anti–PD-1 monotherapy (16). Therefore, we evaluated prognostic value of 20% increase in CX3CR1 score, and found that improved PFS and OS with this cut-off point for chemo-immunotherapy as well (Supplementary Fig. S3). Univariate analysis was performed to compare the characteristics of patients with NSCLC treated with chemo-immunotherapy. This analysis revealed that at least 10% change in CX3CR1 score was the only significant prognostic factors for PFS [HR, 0.28; 95% CI, 0.11–0.75; *P* = 0.011) and OS (HR, 0.25; 95% CI, 0.08–0.80; *P* = 0.019) among other variables such as ECOG status, histology, stage III versus IV, prior chemotherapy, presence of brain metastases, and histologic variables including PD-L1 expression (Table 2). When the factors identified by univariate analysis (*P* < 0.10) were subjected to a multivariate analysis, at least 10% change in CX3CR1 score was the only independent prognostic factor that approached statistical significance for PFS (HR, 0.36; 95% CI, 0.13–1.01; *P* = 0.051), and female sex (HR, 3.115; 95% CI, 1.12–8.67; *P* = 0.030) and at least 10% change in CX3CR1 score (HR, 0.25; 95% CI, 0.07–0.91; *P* = 0.036) were the only independent prognostic factors for OS (Table 2).

**TABLE 2:** Univariate and multivariate logistic regression analyses of demographic and clinical characteristics of patients with NSCLC treated with chemo-immunotherapy

	Progression				Mortality			
	Univariate		Multivariate		Univariate		Multivariate	
Variable	HR (95% CI)	*P*	HR (95% CI)	*P*	HR (95% CI)	*P*	HR (95% CI)	***p*-value**
Age	1.01 (0.96–1.05)	0.793			1.01 (0.96–1.06)	0.710		
Sex								
Female (ref)	2.07				2.59		**3.115**	
Male	(0.83–5.14)	0.117			(0.96–6.98)	0.059	**(1.12–8.67)**	**0.030**
Race								
White (ref)	0.20				0.30			
Black	(0.03–1.52)	0.121			(0.04–2.25)	0.240		
ECOG								
0 (ref)	1.97				2.53		1.29	
1–2	(0.79–4.88)	0.145			(0.94–6.84)	0.067	(0.40–4.20)	0.674
Smoking								
Never (ref)								
Former	0.20 (0.03–1.22)	0.081			0.20 (0.03–1.21)	0.080		
Current	1.02 (0.23–4.52)	0.981			0.65 (0.14–2.94)	0.571		
Histology								
Adenoca. (ref)			1.93					
Squamous cell ca.	2.49 (0.86–7.19)	0.093	(0.64–5.82)	0.245	0.68 (0.39–1.21)	0.189		
Stage								
III (ref)			2.80					
IV	3.47 (0.80–15.1)	0.097	(0.62–12.5)	0.180	4.87 (0.65–36.6)	0.125		
Prior lung surgery								
No (ref)								
Yes	0.929 (0.379–2.28)	0.872			0.73 (0.28–1.89)	0.518		
Prior chemotherapy								
No (ref)								
Yes	1.84 (0.61–5.54)	0.277			1.53 (0.50–4.72)	0.455		
Brain metastases								
No (ref)								
Yes	0.99 (0.33–2.98)	0.985			1.08 (0.62–1.90)	0.784		
PD-L1 Expression								
0%								
1–49%	0.59 (0.20–1.73)	0.338			0.65 (0.21–2.05)	0.463		
≥50%	1.33 (0.46–3.85)	0.597			1.97 (0.64–6.06)	0.239		
Percent change CX3CR1^+^CD8^+^ T cells at 6–9 weeks								
<10% (ref)			0.36					
**≥10%**	**0.28 (0.11–0.75)**	**0.011**	(0.13–1.01)	0.051	**0.25 (0.08–0.80)**	**0.019**	**0.25 (0.07–0.91)**	**0.036**

Abbreviations: Adenoca., adenocarcinoma; ECOG, Eastern Cooperative Oncology Group Performance Status; Squamous cell ca., squamous cell carcinoma.

### Longitudinal Single-cell Profiling of Circulating T Cells in a Patient with NSCLC Treated with Chemo-immunotherapy

Next, we sought to characterize the full spectrum of pre- and early on-treatment circulating T cells early after the initiation of chemo-immunotherapy. We employed scRNA/TCR-seq on serially obtained PBMCs from a patient who received a long-term benefit from chemo-immunotherapy. The patient is a 68-year-old female who was treated with every 3-week carboplatin, pemetrexed, and pembrolizumab for stage IV NSCLC with multiple bilateral pulmonary nodules and mediastinal adenopathy. After two cycles of chemo-immunotherapy, her CT scan at 6 weeks showed an increase in mediastinal and bilateral hilar/infrahilar adenopathy and numerous lung lesions except for a single lesion within the right lower lobe (Supplementary Fig. S4A); however, we found a prompt substantial increase of PB CX3CR1^+^ CD8^+^ T cells and the CX3CR1 score (Supplementary Fig. S4B). Eventually, chemo-immunotherapy was found to be effective with a long-term disease control for 23 months and she lived for longer than 3 years after initiation of the treatment.

**FIGURE 2:**
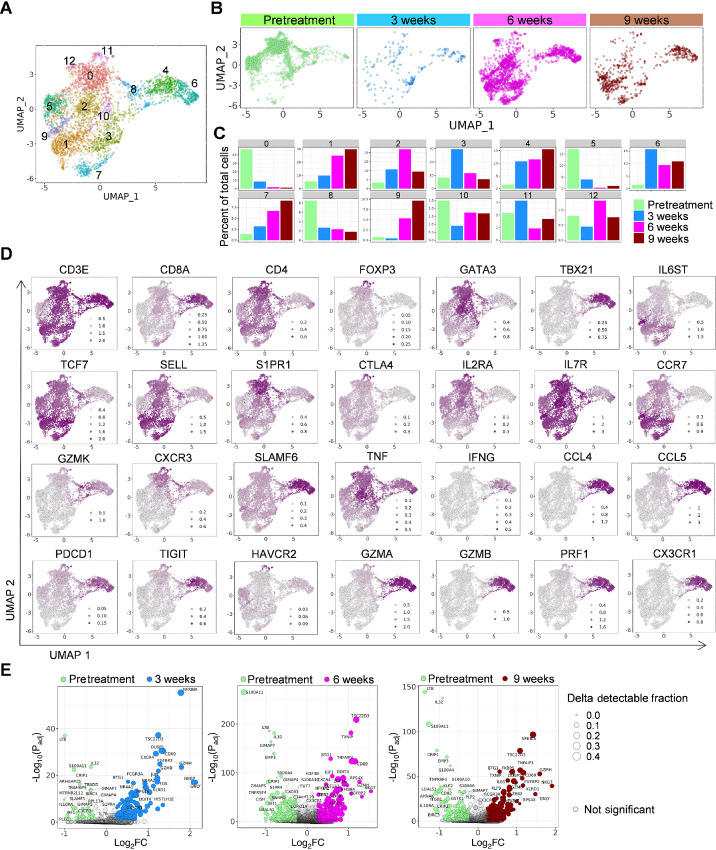
Longitudinal single-cell profiling of circulating T cells in a patient with NSCLC treated with chemo-immunotherapy. **A,** UMAP plots of T-cell subsets in PB. **B** and **C,** UMAP plots (**B**) and frequency of each T-cell cluster (**C**) in PB at different time point as indicated. **D,** Expression plots of indicated genes in PB T-cell clusters. Expression levels are color-coded: gray, not expressed; purple, expressed. **E,** Volcano plots showing enrichment differentially expressed genes in PB T cells at pre- versus during treatment at 3, 6, and 9 weeks. Each green, blue, pink, and brown dot denotes an individual gene with Benjamini–Hochberg-adjusted *P* value < 0.05 and log fold change > 0.25.

To profile PB T cells before and during ICI therapy, we flow-sorted CD45^+^CD19^–^CD3^+^CD56^–^ cells from cryopreserved PBMC samples at pretreatment and 3, 6, and 9 weeks from the initiation of the treatment for scRNA/TCR-seq (Supplementary Fig. S1A and S1B). This yielded data for 4,876 high-quality cells after stringent filtering (pretreatment: 1,861 cells; 3 weeks: 223 cells; 6 weeks: 2,137 cells; and 9 weeks: 655 cells; Supplementary Fig. S1C–S1E). Unsupervised clustering analysis identified 13 distinct lymphocyte clusters [cluster (C)0–12; Fig. 2A; Supplementary Fig. S5; Supplementary Table S4]. Within the clusters expressing *CD8A*, we found a markedly increased frequency of C4- and C6-expressing *CX3CR1* (Fig. 2B–D) consistent with flow cytometric analysis (Supplementary Fig. S4B). Although C6 was notable for high expression levels of *GNLV*, both C4 and C6 overexpressed *NKG7*, *ZEB2*, *GZMB*, *GZMA, GZMH*, and *PRF1* encoding perforin, and genes associated with effector molecules such as *SLAMF6*, *TBX21* encoding T-bet, chemokine (C-C motif) ligands (*CCL4*, *CCL5*), and human leukocyte antigen (HLA) class II molecules, suggesting terminal effector CD8^+^ T cells (Fig. 2D; Supplementary Fig. S5; and Supplementary Table S4). Consistent with this, these clusters were notable for high levels of TCR, PD-1, IFN, proliferative, and cytokine-mediated signaling pathways (Supplementary Fig. S6; Supplementary Table S5A and S5B). There was a gradual increase of C7-expressing markers of memory T cells, *TCF7* encoding T-cell factor 1 (TCF1), *SELL* encoding L-selectin, *IL7R* and *CCR7*. In contrast, there was a decrease of C8 that was notable for elevated expression of *GZMK* and *CXCR3*. C8 was also positive for genes related to activated (*PDCD1* encoding PD-1, *IL2RA*), effector (*NKG7*, *GZMA*, *GZMM*, *CCL5*,) and memory (*TCF7*, *SELL*, *IL7R*, *CCR7*) T cells, but relatively negative for *CX3CR1* expression, suggesting activated early effector T cells. C0 expressing *CD4*, *TCF7*, *SELL*, *S1PR1*, *GATA3*, and *IL7R* was the most frequent cluster at pretreatment, but considerably decreased during chemo-immunotherapy. Although this cluster was enriched with TNF-mediated signaling pathway, it also exhibited high levels of apoptosis and programmed cell death pathways. In line with these changes in specific cluster frequencies, differential expression analysis identified that genes associated with terminal differentiation and effector function including *CX3CR1*, *NKG7*, *GNLY*, *GZMB*, *GZMH*, *CCL4*, *CCL5* in circulating T cells were markedly increased as early as 3 weeks from the initiation of chemo-immunotherapy (Fig. 2E; Supplementary Table S6A–S6C). Taken together, effective chemo-immunotherapy induces dynamic changes in the composition of circulating T cells early on-treatment including increased frequency of terminal effector *CX3CR1*^+^ CD8^+^ T cells.

### TIL Repertoires Expanding in PB are Terminal Effector T Cells Expressing *CX3CR1*

**FIGURE 3:**
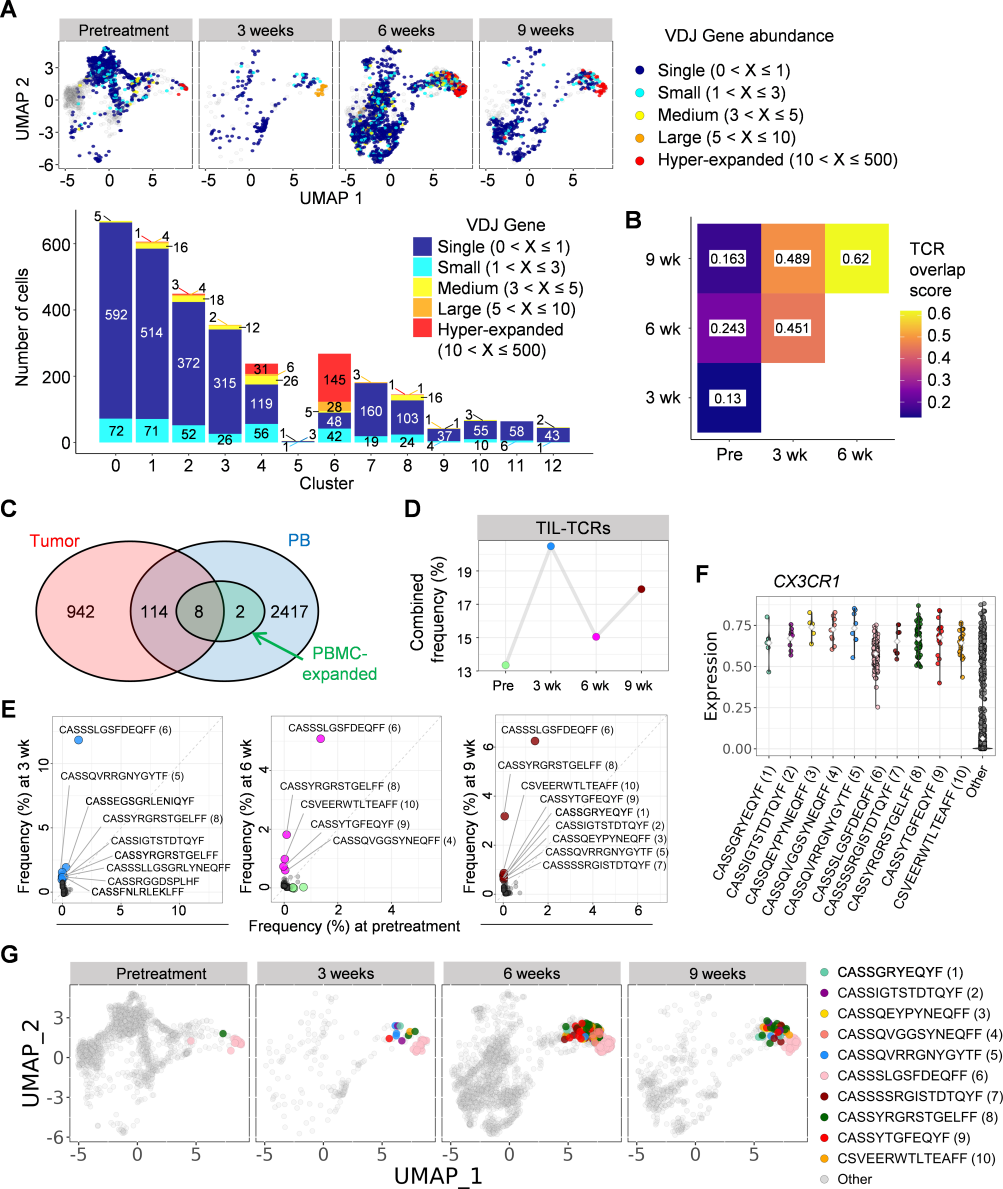
TIL repertoires expanding in PB are terminal effector T cells expressing *CX3CR1*. **A,** UMAP plots (top) and frequency of expanding T cells in each T-cell cluster (bottom) in PB. **B,** TCR rearrangement gene overlap by Morisita index between PB samples. **C,** Venn diagram of the number of T-cell repertoires in tumors identified by TCRβ sequencing and PB identified by scRNA/TCR-seq. **D,** Combined frequency of circulating TIL repertoires (TIL-TCRs) at different time points as indicated. Frequent TIL-TCRs were identified as productive TCRβ clonotypes with at least 5 counts by TCRβ sequencing of pretreatment archival tumor samples as shown in Supplementary Fig. S7. **E,** Identification of clonally expanded T-cell repertoires early on-treatment at 3, 6, and 9 weeks versus pre-treatment. **F,***CX3CR1* expression in circulating clonally expanded CD8^+^ T cells at 6 and 9 weeks. **G,** UMAP plots of circulating clonally expanded CD8^+^ T cells at different time points as indicated.

We have recently shown that successful ICI therapy induces an expansion of the peripheral CX3CR1^+^ CD8^+^ T-cell subset that includes an enriched repertoire of tumor-specific and tumor-infiltrating CD8^+^ T cells in preclinical models (16). To evaluate these findings in this patient (Supplementary Fig. S4), we first assessed VDJ gene usage in the PB scRNA/TCR-seq data. This analysis identified 2,541 unique productive TCRB clonotypes, and revealed a higher concentration of larger clonally expanded T cells in C4 and C6 (Fig. 3A). Second, analysis of T-cell repertoire similarities amongst samples revealed a dramatic reshaping of T-cell repertoires, demonstrated by lower TCR overlap score occurring within 3 weeks of treatment (Fig. 3B). Finally, we sought to determine whether circulating clonally expanded T cells could be identified in the tumor. To this end, we extracted DNA from the archival pretreatment tumor samples, and performed TCR-seq to identify TCR clonotypes observed in the tumor (TIL-TCRs). We identified 16,109 unique productive TCRB clonotypes (Supplementary Table S7), of which 1,064 (6.6%) were observed at least 5 counts in sequencing, and were classified as frequent TIL-TCRs (Supplementary Fig. S7). Of these, 122 (11.5%) TIL-TCRs were identified in PB scRNA/TCR-seq data (Fig. 3C). The combined frequency of these TIL-TCRs was increased in the periphery after the initiation of chemo-immunotherapy (Fig. 3D; Supplementary Table S8). The scRNA/TCR-seq analysis identified 10 significantly expanded clonotypes in PB at 6 and 9 weeks compared with pretreatment (Fig. 3E; Supplementary Table S9A–C), including 8 frequent TIL-TCRs (Fig. 3C; Supplementary Table S9). All 10 circulating expanded clones overexpressed *CX3CR1* (Fig. 3F), and were enriched in C4 and C6 (Fig. 3G). Collectively, these findings suggest that circulating clonally expanded T cells early after the initiation of chemo-immunotherapy overexpress *CX3CR1*, and contain repertoires of tumor-infiltrating T cells.

## Discussion

In this study, using prospectively collected longitudinal blood samples, we provide evidence that effective combination chemo-immunotherapy correlates with an early on-treatment expansion of circulating CX3CR1^+^ CD8^+^ T cells. The increase of CX3CR1 score, regardless of whether it was 10% or 20%, was associated with both increased PFS and OS. Our results are consistent with previous findings in patients with renal cell carcinoma and NSCLC treated with PD-1/PD-L1 blockade monotherapy, and patients with melanoma undergoing chemo-immunotherapy (16, 22, 34). Our findings further demonstrated that at least 10% increase of the CX3CR1 score was predictive of response to chemo-immunotherapy with high sensitivity, specificity, PPV, and NPV, compared with pretreatment PD-L1 expression on immune and tumor cells. The CX3CR1 score was predictive of response to anti–PD-1 monotherapy (16) and chemo-immunotherapy in this study; however, the optimal cut-off score was different: 20% for anti–PD-1 monotherapy (15) and 10% for chemo-immunotherapy. It is possible that the chemotherapy component of treatment might have affected T-cell differentiation although direct evidence for this explanation has been lacking. While beneficial immunologic effects of chemotherapy such as induction of immunogenic cell death and depletion of immunosuppressor cells have been recognized (35), chemotherapy could affect proliferation of antitumor T cells induced by anti–PD-1 therapy (36, 37). Additional work is needed to evaluate the impact of chemotherapy on differentiation and cytotoxic function of effector T cells rescued by ICI therapy.

The potential value of an increase in the CX3CR1 score following initiation of therapy was illustrated by a patient who was not considered to be a responder based on the serial imaging but received long-term benefit from chemo-immunotherapy. In this patient, consistent with a remarkable expansion of PB CX3CR1^+^ CD8^+^ T cells, single-cell profiling of longitudinal circulating T cells revealed effective early on-treatment differentiation and expansion of effector T cells that includes tumor resident T-cell repertoires. Moreover, we found a notable reshaping of the circulating TCR clonotypes 3 weeks after initiation of the treatment. These findings suggest that evolution of the immune landscape observed in the TME during ICI therapy (38, 39) could be identified in circulating immune cells, and might be useful for treatment decision-making.

Combining scRNA/TCR-seq of sorted T cells in serial PB samples with TCR-seq of tumor tissue provided insight into the transcriptional landscape of circulating TIL-TCRs, and revealed that expanded circulating TIL-TCRs early on-treatment were enriched in signatures of terminal effector differentiation and marked by high expression of *CX3CR1*. While the data were obtained from a single patient, these findings are in agreement with our recent study showing that peripheral CX3CR1^+^ CD8^+^ T-cell clones reflect the TCR repertoires in CD8^+^ TILs in preclinical models (15), suggesting that T-cell CX3CR1 expression may act as a dynamic blood-based biomarker of response to immunotherapy. Of note, TIL-TCRs expanded in response to chemo-immunotherapy could be identified within the same cluster expressing *CX3CR1* at pretreatment in line with recent studies evaluating the tumor and a time-matched blood sample from patients with melanoma with scRNA/TCR-seq (21, 40). However, this was markedly pronounced in early on-treatment PB samples in this study.

Our study has several limitations. While our findings on CX3CR1 score are statistically significant, our cohort is relatively small, resulting in broad confidence intervals. On the basis of Cox univariate analysis, at least 10% change in CX3CR1 score was the only significant predictor of PFS and OS; however, the lack of effect of other key variables such as stage III versus IV and brain metastasis almost certainly reflects the limited power of our small cohort. To validate our results, follow up studies are necessary in larger sample sizes and with independent cohorts. There was some variability in our chemo-immune regimens, with three different regimens in NSCLC employed across patients, and the number of patients was underpowered to detect any difference in outcome between the different regimens. Our cohort did not include patients who had radiotherapy during chemo-immunotherapy, and therefore it remains unclear how the CX3CR1 score would behave in this patient subset. Because of the limited availability of tumor tissue for TCR-seq, single-cell profiling of circulating TIL-TCRs was limited to a single patient in the current cohort. In addition, intratumor heterogeneity of TIL-TCRs might have not been fully captured because DNA was not extracted from the entire tumor for TCR-seq. It remains unclear whether clonally expanded TIL-TCRs in PB also did in the tumor during chemo-immunotherapy because on-treatment tumor tissue was not available in this patient. More studies are needed to elucidate further genomic and transcriptomic characterization of TIL-TCRs responding to immunotherapy.

Given the curative potential of the treatment and the evolution of the TME during ICI therapy (38, 39), early on-treatment blood-based biomarkers with high NPV would be of great value (41). Because ICI therapy targets host immunity, investigating the potential utility of markers expressed on circulating immune cells in clinical applications as a predictive biomarker has been an intense area of research. There are several mechanistic advantages to the use of CX3CR1 as a biomarker in this regard due to (i) the unidirectional differentiation of CX3CR1^int^ to CX3CR1^hi^ subsets, allowing CX3CR1 to be stably expressed on CD8^+^ T cells (13–15); and (ii) the decreased expression of L-selectin and CXCR3 (13–16), essential trafficking molecules for T cells from blood to secondary lymphoid organs and the TME, respectively (18–20), allowing CX3CR1^+^ CD8^+^ T cells to remain in circulation at the end of the primary response (13, 15).

In summary, our findings demonstrate that at least 10% increase of the CX3CR1^+^ subset in PB CD8^+^ T cells identifies patients with NSCLC responding to chemo-immunotherapy early on-treatment. Substantial early on-treatment reshaping of T-cell clonotypes and clonally expanded TIL-TCRs expressing *CX3CR1* can be identified in PB of patients undergoing chemo-immunotherapy. Our study provides evidence to plan further trials testing this circulating T-cell differentiation marker in larger prospective trials in a variety of malignancies.

## Supplementary Material

Supplementary Tables S1-9 Supplementary Tables 1-9

Supplementary Figure S1 Supplementary Figure 1: Single-cell RNA/TCR sequencing (scRNA/TCR-seq) quality assessment. A Gating strategy of identifying single live CD45+CD19–CD3+CD56– cells from cryopreserved peripheral mononuclear blood cells for scRNA/TCR-seq. B Representative flow cytometric plots showing the frequency of CD45+CD19–CD3+CD56– cells after flow-sort. C Scatterplots depicting total features, counts, doublet scores, mitochondrial content and ribosomal content across all cells prior to filtering. Filtering thresholds applied are shown. D Elbow plot of PCA (top 50 components). PC selection for downstream analysis was limited to those components accounting for at least 0.1 % of total variation (n = 44). E UMAP representations of all-post filtered, normalized cells showing cellular distributions of total unique counts, mitochondrial scores, ribosomal scores, G2M/S phase scores and doublet scores. F UMAP plot identifying doublets by hashtag oligos (HTO).

Supplementary Figure S2 Supplementary Figure 2. Baseline (high vs. low) frequency of peripheral blood (PB) CX3CR1+ CD8+ T cells does not associate with response and prognosis in NSCLC patients undergoing chemo-immunotherapy. Related to Fig. 1 Overall response rate (ORR), progression free survival (PFS), and overall survival (OS) of patients with high and low pre-treatment frequency of the CX3CR1+ subset in PB CD8+ T cells at various cut-off points. P values were calculated by a log-rank (Mantel-Cox) test.

Supplementary Figure S3 Supplementary Figure 3. An increase of the CX3CR1 score is associated with better survival in NSCLC patients undergoing chemo-immunotherapy. Related to Fig. 1E Progression free survival (PFS) and overall survival (OS) for high (≥ 20%) versus low (< 20%) CX3CR1 score. P values were calculated by a log-rank (Mantel-Cox) test.

Supplementary Figure S4 Supplementary Figure 4. Related to Fig. 2, 3 and Supplementary Fig. 5-7. 68-year-old female with bilateral lung metastases and multiple mediastinal lymph node metastasis was treated with chemo-immunotherapy (carboplatin, pemetrexed, and pembrolizumab). PD-L1 expression in the pre-treatment tumor specimen was 2%. The patient had stable disease for 621 days with overall survival > 3 years. A Contrast-enhanced cross-sectional imaging obtained at prior to and during treatment. B Expression of CX3CR1 in peripheral blood CD8 T cells (left) and the CX3CR1 score (right) at different time points as indicated.

Supplementary Figure S5 Supplementary Figure 5. Related to Figure 2 and Supplementary Table 4. Analysis of peripheral blood CD45+CD19–CD3+CD56– cells by scRNA/TCR-seq. Heatmap of all cells showing the expression levels of the 10 most discriminative genes per cell type (in rows) across all the identified cell populations (in columns). Gene expression in each clusters are also listed in Supplementary Table 4. Color-code layout: scale of purple to yellow; from lowest expression to highest expression.

Supplementary Figure S6 Supplementary Figure 6. Related to Fig. 2 and Supplementary Table 5A, B. Heat map showing the top 10 top significantly enriched pathways found in each T-cell cluster. Gene sets from Reactome (A) and Gene Ontology-Biological Processes (GO-BP) (B) are shown separately. Only gene sets with Benjamini-Hochberg-adjusted p < 0.05 were considered as significantly enriched.

Supplementary Figure S7 Supplementary Figure 7. Related to Fig. 3 Bar graphs displaying the counts of tumor-infiltrating lymphocytes (TILs) expressing a given TCRβ clonotype on the y axis and individual TIL clonotypes ordered by increasing frequency on the x axis for a patient in supplementary figure 4.

## References

[1] Reck M , Rodríguez-Abreu D, Robinson AG, Hui R, Csőszi T, Fülöp A, . Pembrolizumab versus chemotherapy for PD-L1-positive non-small-cell lung cancer. N Engl J Med 2016;375:1823–33. 27718847 10.1056/NEJMoa1606774

[2] Herbst RS , Giaccone G, de Marinis F, Reinmuth N, Vergnenegre A, Barrios CH, . Atezolizumab for first-line treatment of PD-L1-selected patients with NSCLC. N Engl J Med 2020;383:1328–39. 32997907 10.1056/NEJMoa1917346

[3] Mok TSK , Wu YL, Kudaba I, Kowalski DM, Cho BC, Turna HZ, . Pembrolizumab versus chemotherapy for previously untreated, PD-L1-expressing, locally advanced or metastatic non-small-cell lung cancer (KEYNOTE-042): a randomised, open-label, controlled, phase 3 trial. Lancet 2019;393:1819–30. 30955977 10.1016/S0140-6736(18)32409-7

[4] Hellmann MD , Paz-Ares L, Caro RB, Zurawski B, Kim SW, Costa EC, . Nivolumab plus ipilimumab in advanced non-small-cell lung cancer. N Engl J Med 2019;381:2020–31. 31562796 10.1056/NEJMoa1910231

[5] National Comprehensive Cancer Network. Non-small cell lung cancer, version 1.2021; 2020. Available from: https://www.nccn.org/professionals/physician_gls.

[6] Paz-Ares L , Luft A, Vicente D, Tafreshi A, Gümüş M, Mazières J, . Pembrolizumab plus chemotherapy for squamous non-small-cell lung cancer. N Engl J Med 2018;379:2040–51. 30280635 10.1056/NEJMoa1810865

[7] Langer CJ , Gadgeel SM, Borghaei H, Papadimitrakopoulou VA, Patnaik A, Powell SF, . Carboplatin and pemetrexed with or without pembrolizumab for advanced, non-squamous non-small-cell lung cancer: a randomised, phase 2 cohort of the open-label KEYNOTE-021 study. Lancet Oncol 2016;17:1497–508. 27745820 10.1016/S1470-2045(16)30498-3 PMC6886237

[8] West H , McCleod M, Hussein M, Morabito A, Rittmeyer A, Conter HJ, . Atezolizumab in combination with carboplatin plus nab-paclitaxel chemotherapy compared with chemotherapy alone as first-line treatment for metastatic non-squamous non-small-cell lung cancer (IMpower130): a multicentre, randomised, open-label, phase 3 trial. Lancet Oncol 2019;20:924–37. 31122901 10.1016/S1470-2045(19)30167-6

[9] Herbst RS , Baas P, Kim DW, Felip E, Perez-Gracia JL, Han JY, . Pembrolizumab versus docetaxel for previously treated, PD-L1-positive, advanced non-small-cell lung cancer (KEYNOTE-010): a randomised controlled trial. Lancet 2016;387:1540–50. 26712084 10.1016/S0140-6736(15)01281-7

[10] Bodor JN , Boumber Y, Borghaei H. Biomarkers for immune checkpoint inhibition in non-small cell lung cancer (NSCLC). Cancer 2020;126:260–70. 31691957 10.1002/cncr.32468 PMC7372560

[11] Puzanov I , Diab A, Abdallah K, Bingham CO, Brogdon C, Dadu R, . Managing toxicities associated with immune checkpoint inhibitors: consensus recommendations from the society for immunotherapy of cancer (SITC) toxicity management working group. J Immunother Cancer 2017;5:95. 29162153 10.1186/s40425-017-0300-z PMC5697162

[12] Imai T , Hieshima K, Haskell C, Baba M, Nagira M, Nishimura M, . Identification and molecular characterization of fractalkine receptor CX3CR1, which mediates both leukocyte migration and adhesion. Cell 1997;91:521–30. 9390561 10.1016/s0092-8674(00)80438-9

[13] Gerlach C , Moseman EA, Loughhead SM, Alvarez D, Zwijnenburg AJ, Waanders L, . The chemokine receptor CX3CR1 defines three antigen-experienced CD8 T cell subsets with distinct roles in immune surveillance and homeostasis. Immunity 2016;45:1270–84. 27939671 10.1016/j.immuni.2016.10.018 PMC5177508

[14] Gordon CL , Lee LN, Swadling L, Hutchings C, Zinser M, Highton AJ, . Induction and maintenance of CX3CR1-intermediate peripheral memory CD8(+) T cells by persistent viruses and vaccines. Cell Rep 2018;23:768–82. 29669283 10.1016/j.celrep.2018.03.074 PMC5917822

[15] Yamauchi T , Hoki T, Oba T, Saito H, Attwood K, Sabel MS, . CX3CR1-CD8+ T cells are critical in antitumor efficacy, but functionally suppressed in the tumor microenvironment. JCI insight 2020;5:e133920. 32255766 10.1172/jci.insight.133920 PMC7205436

[16] Yamauchi T , Hoki T, Oba T, Jain V, Chen H, Attwood K, . T-cell CX3CR1 expression as a dynamic blood-based biomarker of response to immune checkpoint inhibitors. Nat Commun 2021;12:1402. 33658501 10.1038/s41467-021-21619-0 PMC7930182

[17] Yamauchi T , Hoki T, Oba T, Kajihara R, Attwood K, Cao X, . CD40 and CD80/86 signaling in cDC1s mediate effective neoantigen vaccination and generation of antigen-specific CX3CR1(+) CD8(+) T cells. Cancer Immunol Immunother 2021;71:137–151. 34037810 10.1007/s00262-021-02969-6 PMC8715856

[18] Mikucki ME , Fisher DT, Matsuzaki J, Skitzki JJ, Gaulin NB, Muhitch JB, . Non-redundant requirement for CXCR3 signalling during tumoricidal T-cell trafficking across tumour vascular checkpoints. Nat Commun 2015;6:7458. 26109379 10.1038/ncomms8458 PMC4605273

[19] von Andrian UH , Mempel TR. Homing and cellular traffic in lymph nodes. Nat Rev Immunol 2003;3:867–78. 14668803 10.1038/nri1222

[20] Gallatin WM , Weissman IL, Butcher EC. A cell-surface molecule involved in organ-specific homing of lymphocytes. Nature 1983;304:30–4. 6866086 10.1038/304030a0

[21] Pauken KE , Shahid O, Lagattuta KA, Mahuron KM, Luber JM, Lowe MM, . Single-cell analyses identify circulating anti-tumor CD8 T cells and markers for their enrichment. J Exp Med 2021;218:e20200920. 33651880 10.1084/jem.20200920 PMC7933992

[22] Yan Y , Cao S, Liu X, Harrington SM, Bindeman WE, Adjei AA, . CX3CR1 identifies PD-1 therapy-responsive CD8+ T cells that withstand chemotherapy during cancer chemoimmunotherapy. JCI insight 2018;3:e97828. 29669928 10.1172/jci.insight.97828 PMC5931117

[23] Oba T , Long MD, Keler T, Marsh HC, Minderman H, Abrams SI, . Overcoming primary and acquired resistance to anti-PD-L1 therapy by induction and activation of tumor-residing cDC1s. Nat Commun 2020;11:5415. 33110069 10.1038/s41467-020-19192-z PMC7592056

[24] Sauerbrei W , Taube SE, McShane LM, Cavenagh MM, Altman DG. Reporting recommendations for tumor marker prognostic studies (REMARK): an abridged explanation and elaboration. J Natl Cancer Inst 2018;110:803–11. 29873743 10.1093/jnci/djy088 PMC6093349

[25] Seymour L , Bogaerts J, Perrone A, Ford R, Schwartz LH, Mandrekar S, . iRECIST: guidelines for response criteria for use in trials testing immunotherapeutics. Lancet Oncol 2017;18:e143-52. 28271869 10.1016/S1470-2045(17)30074-8 PMC5648544

[26] Dako. PD-L1 IHC 28–8 pharmDx: non-squamous non-small cell lung cancer [interpretation manual]. Santa Clara, CA: Dako; 2017.

[27] Butler A , Hoffman P, Smibert P, Papalexi E, Satija R. Integrating single-cell transcriptomic data across different conditions, technologies, and species. Nat Biotechnol 2018;36:411–20. 29608179 10.1038/nbt.4096 PMC6700744

[28] Makino K , Long MD, Kajihara R, Matsueda S, Oba T, Kanehira K, . Generation of cDC-like cells from human induced pluripotent stem cells via Notch signaling. J Immunother Cancer 2022;10:e003827. 35101945 10.1136/jitc-2021-003827 PMC8804689

[29] Borcherding N , Bormann NL, Kraus G. scRepertoire: an R-based toolkit for single-cell immune receptor analysis. F1000Res 2020;9:47. 32789006 10.12688/f1000research.22139.1 PMC7400693

[30] Xie Z , Bailey A, Kuleshov MV, Clarke DJB, Evangelista JE, Jenkins SL, . Gene set knowledge discovery with enrichr. Curr Protoc 2021;1:e90. 33780170 10.1002/cpz1.90 PMC8152575

[31] Liberzon A , Subramanian A, Pinchback R, Thorvaldsdóttir H, Tamayo P, Mesirov JP. Molecular signatures database (MSigDB) 3.0. Bioinformatics 2011;27:1739–40. 21546393 10.1093/bioinformatics/btr260 PMC3106198

[32] DeLong ER , DeLong DM, Clarke-Pearson DL. Comparing the areas under two or more correlated receiver operating characteristic curves: a nonparametric approach. Biometrics 1988;44:837–45. 3203132

[33] Youden WJ . Index for rating diagnostic tests. Cancer 1950;3:32–5. 15405679 10.1002/1097-0142(1950)3:1<32::aid-cncr2820030106>3.0.co;2-3

[34] Wallin JJ , Bendell JC, Funke R, Sznol M, Korski K, Jones S, . Atezolizumab in combination with bevacizumab enhances antigen-specific T-cell migration in metastatic renal cell carcinoma. Nat Commun 2016;7:12624. 27571927 10.1038/ncomms12624 PMC5013615

[35] Galluzzi L , Humeau J, Buqué A, Zitvogel L, Kroemer G. Immunostimulation with chemotherapy in the era of immune checkpoint inhibitors. Nat Rev Clin Oncol 2020;17:725–41. 32760014 10.1038/s41571-020-0413-z

[36] Das RK , O'Connor RS, Grupp SA, Barrett DM. Lingering effects of chemotherapy on mature T cells impair proliferation. Blood 2020;4:4653–64. 10.1182/bloodadvances.2020001797 PMC7556159 33002133

[37] Kamphorst AO , Pillai RN, Yang S, Nasti TH, Akondy RS, Wieland A, . Proliferation of PD-1+ CD8 T cells in peripheral blood after PD-1-targeted therapy in lung cancer patients. Proc Natl Acad Sci U S A 2017;114:4993–8. 28446615 10.1073/pnas.1705327114 PMC5441721

[38] Riaz N , Havel JJ, Makarov V, Desrichard A, Urba WJ, Sims JS, . Tumor and microenvironment evolution during immunotherapy with nivolumab. Cell 2017;171:934–49.e16. 29033130 10.1016/j.cell.2017.09.028 PMC5685550

[39] Vilain RE , Menzies AM, Wilmott JS, Kakavand H, Madore J, Guminski A, . Dynamic changes in PD-L1 expression and immune infiltrates early during treatment predict response to PD-1 blockade in melanoma. Clin Cancer Res 2017;23:5024–33. 28512174 10.1158/1078-0432.CCR-16-0698

[40] Lucca LE , Axisa PP, Lu B, Harnett B, Jessel S, Zhang L, . Circulating clonally expanded T cells reflect functions of tumor-infiltrating T cells. J Exp Med 2021;218:e20200921. 33651881 10.1084/jem.20200921 PMC7933991

[41] Lesterhuis WJ , Bosco A, Millward MJ, Small M, Nowak AK, Lake RA. Dynamic versus static biomarkers in cancer immune checkpoint blockade: unravelling complexity. Nat Rev Drug Discov 2017;16:264–72. 28057932 10.1038/nrd.2016.233

