# Supplementary material for: Predictive and Prognostic Implications of Circulating CX3CR1+ CD8+ T Cells in Non–Small Cell Lung Cancer Patients Treated with Chemo-Immunotherapy
Source: Cancer Res Commun. 2023 Mar 30;3(3):510–20. doi: 10.1158/2767-9764.CRC-22-0383 (PMC10060186; doi:10.1158/2767-9764.CRC-22-0383)
Supplement: Supplementary Figure S1 — Supplementary Figure 1: Single-cell RNA/TCR sequencing (scRNA/TCR-seq) quality assessment. A Gating strategy of identifying single live CD45+CD19–CD3+CD56– cells from cryopreserved peripheral mononuclear blood cells for scRNA/TCR-seq. B Representative flow cytometric plots showing the frequency of CD45+CD19–CD3+CD56– cells after flow-sort. C Scatterplots depicting total features, counts, doublet scores, mitochondrial content and ribosomal content across all cells prior to filtering. Filtering thresholds applied are shown. D Elbow plot of PCA (top 50 components). PC selection for downstream analysis was limited to those components accounting for at least 0.1 % of total variation (n = 44). E UMAP representations of all-post filtered, normalized cells showing cellular distributions of total unique counts, mitochondrial scores, ribosomal scores, G2M/S phase scores and doublet scores. F UMAP plot identifying doublets by hashtag oligos (HTO). [file crc-22-0383-s02.pdf]

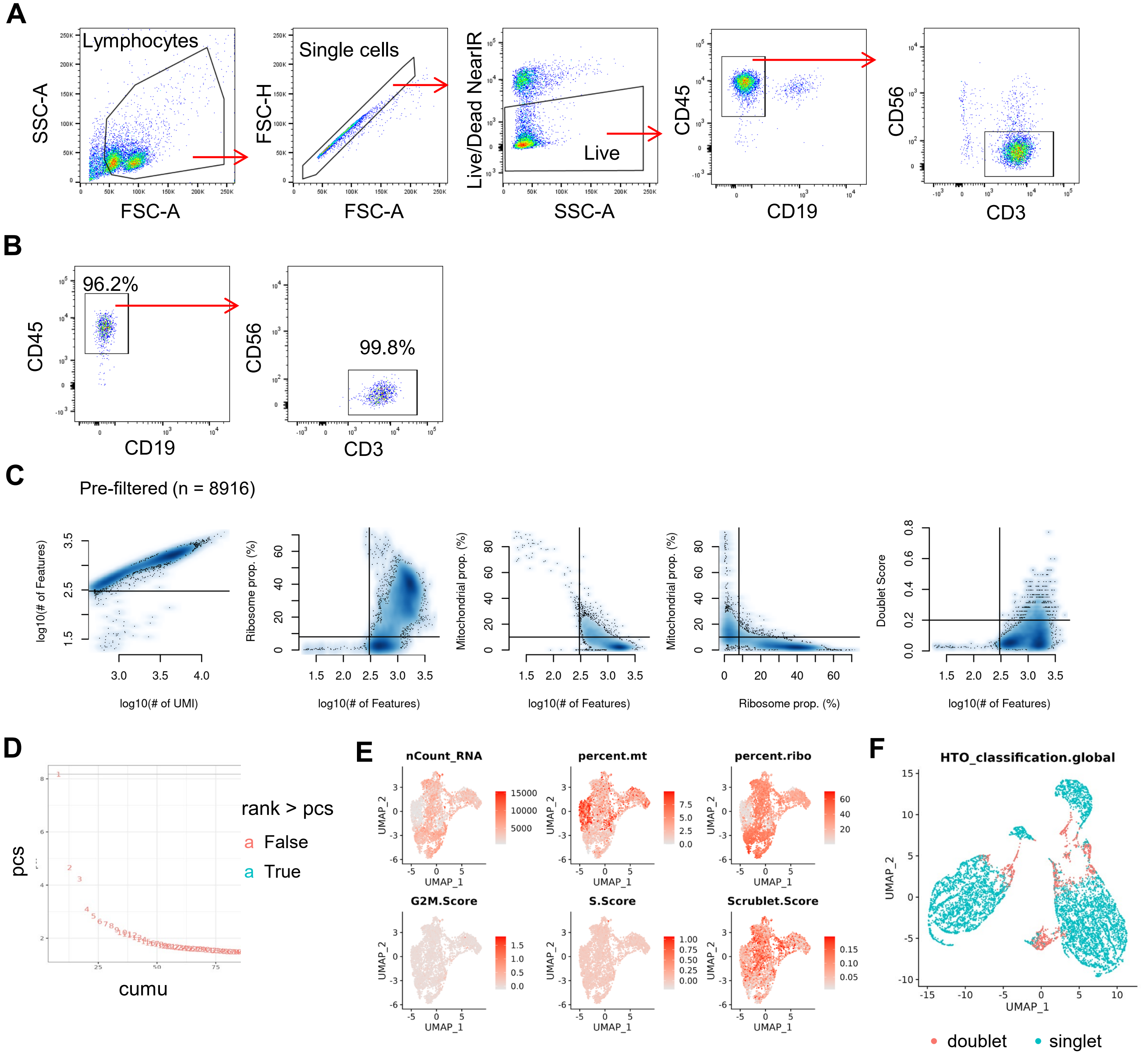

Supplementary Figure 1: Single-cell RNA/TCR sequencing (scRNA/TCR-seq) quality assessment.
A Gating strategy of identifying single live $CD45^{+}CD19^{-}CD3^{+}CD56^{-}$ cells from cryopreserved peripheral mononuclear blood cells for scRNA/TCR-seq.
B Representative flow cytometric plots showing the frequency of $CD45^{+}CD19^{-}CD3^{+}CD56^{-}$ cells after flow-sort.
C Scatterplots depicting total features, counts, doublet scores, mitochondrial content and ribosomal content across all cells prior to filtering. Filtering thresholds applied are shown.
D Elbow plot of PCA (top 50 components). PC selection for downstream analysis was limited to those components accounting for at least 0.1 % of total variation (n = 44).
E UMAP representations of all-post filtered, normalized cells showing cellular distributions of total unique counts, mitochondrial scores, ribosomal scores, G2M/S phase scores and doublet scores.
F UMAP plot identifying doublets by hashtag oligos (HTO).
