# Supplementary material for: Predictive and Prognostic Implications of Circulating CX3CR1+ CD8+ T Cells in Non–Small Cell Lung Cancer Patients Treated with Chemo-Immunotherapy
Source: Cancer Res Commun. 2023 Mar 30;3(3):510–20. doi: 10.1158/2767-9764.CRC-22-0383 (PMC10060186; doi:10.1158/2767-9764.CRC-22-0383)
Supplement: Supplementary Figure S2 — Supplementary Figure 2. Baseline (high vs. low) frequency of peripheral blood (PB) CX3CR1+ CD8+ T cells does not associate with response and prognosis in NSCLC patients undergoing chemo-immunotherapy. Related to Fig. 1 Overall response rate (ORR), progression free survival (PFS), and overall survival (OS) of patients with high and low pre-treatment frequency of the CX3CR1+ subset in PB CD8+ T cells at various cut-off points. P values were calculated by a log-rank (Mantel-Cox) test. [file crc-22-0383-s03.pdf]

Suppl. Fig. 2

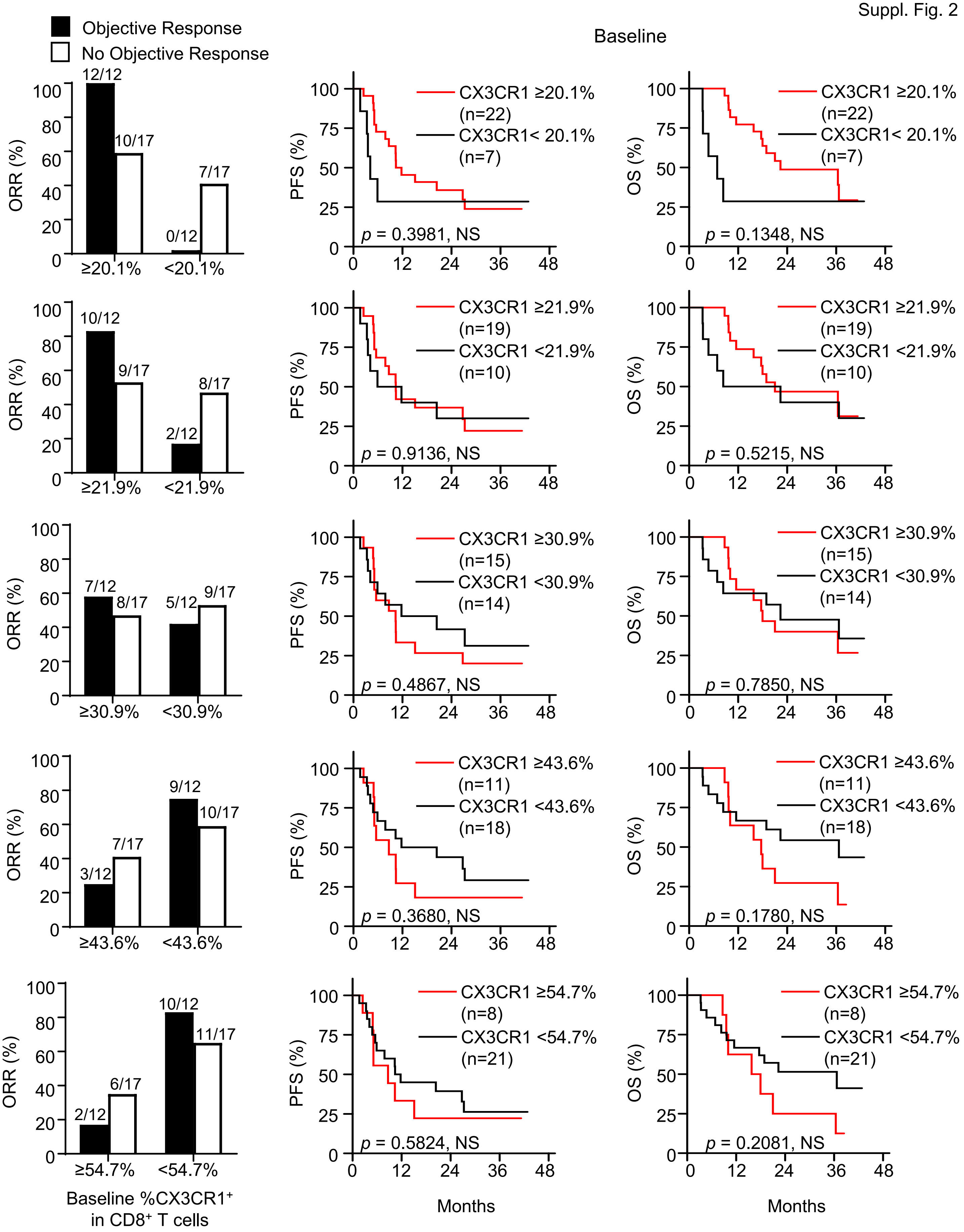

Supplementary Figure 2. Baseline (high vs. low) frequency of peripheral blood (PB) CX3CR1+ CD8+ T cells does not associate with response and prognosis in NSCLC patients undergoing chemo-immunotherapy. Related to Fig. 1

Overall response rate (ORR), progression free survival (PFS), and overall survival (OS) of patients with high and low pre-treatment frequency of the CX3CR1+ subset in PB CD8+ T cells at various cut-off points. *P* values were calculated by a log-rank (Mantel-Cox) test.
