# Supplementary material for: Predictive and Prognostic Implications of Circulating CX3CR1+ CD8+ T Cells in Non–Small Cell Lung Cancer Patients Treated with Chemo-Immunotherapy
Source: Cancer Res Commun. 2023 Mar 30;3(3):510–20. doi: 10.1158/2767-9764.CRC-22-0383 (PMC10060186; doi:10.1158/2767-9764.CRC-22-0383)
Supplement: Supplementary Figure S3 — Supplementary Figure 3. An increase of the CX3CR1 score is associated with better survival in NSCLC patients undergoing chemo-immunotherapy. Related to Fig. 1E Progression free survival (PFS) and overall survival (OS) for high (≥ 20%) versus low (< 20%) CX3CR1 score. P values were calculated by a log-rank (Mantel-Cox) test. [file crc-22-0383-s04.pdf]

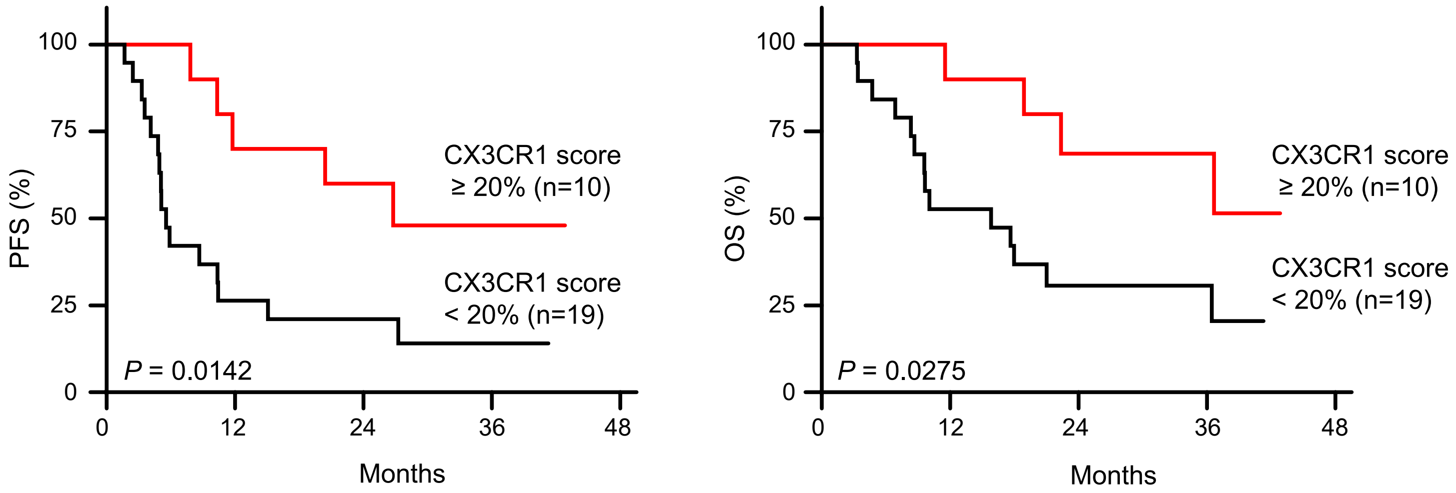

Supplementary Figure 3. An increase of the CX3CR1 score is associated with better survival in NSCLC patients undergoing chemo-immunotherapy. Related to Fig. 1E

Progression free survival (PFS) and overall survival (OS) for high (≥ 20%) versus low (< 20%) CX3CR1 score. *P* values were calculated by a log-rank (Mantel-Cox) test.
