# Supplementary material for: Predictive and Prognostic Implications of Circulating CX3CR1+ CD8+ T Cells in Non–Small Cell Lung Cancer Patients Treated with Chemo-Immunotherapy
Source: Cancer Res Commun. 2023 Mar 30;3(3):510–20. doi: 10.1158/2767-9764.CRC-22-0383 (PMC10060186; doi:10.1158/2767-9764.CRC-22-0383)
Supplement: Supplementary Figure S4 — Supplementary Figure 4. Related to Fig. 2, 3 and Supplementary Fig. 5-7. 68-year-old female with bilateral lung metastases and multiple mediastinal lymph node metastasis was treated with chemo-immunotherapy (carboplatin, pemetrexed, and pembrolizumab). PD-L1 expression in the pre-treatment tumor specimen was 2%. The patient had stable disease for 621 days with overall survival > 3 years. A Contrast-enhanced cross-sectional imaging obtained at prior to and during treatment. B Expression of CX3CR1 in peripheral blood CD8 T cells (left) and the CX3CR1 score (right) at different time points as indicated. [file crc-22-0383-s05.pdf]

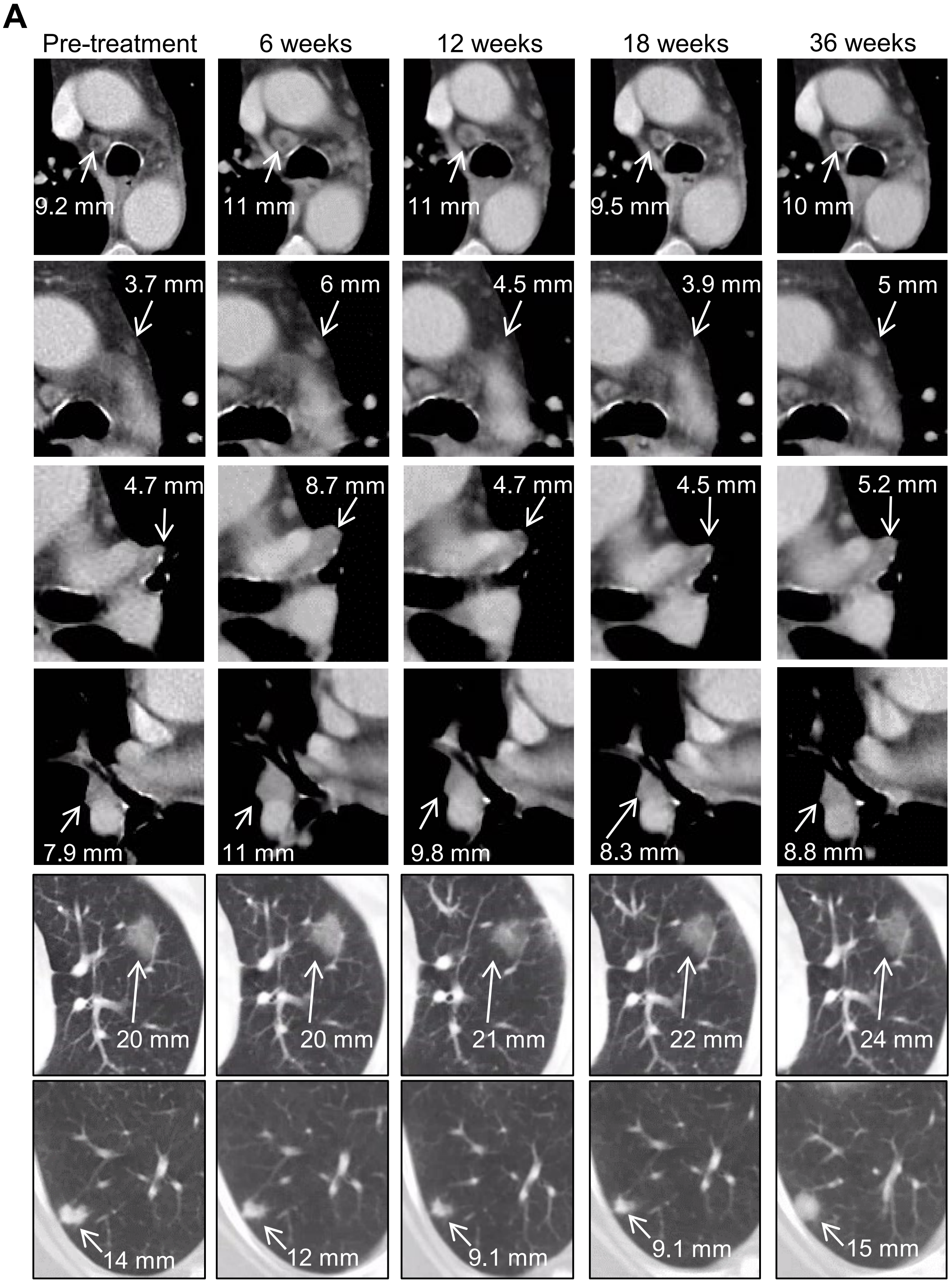

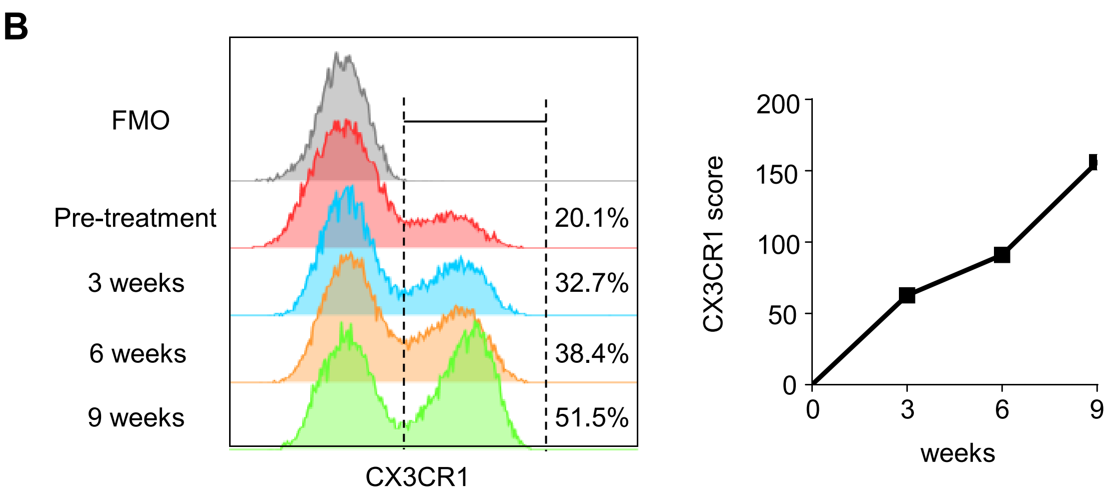

Supplementary Figure 4. Related to Fig. 2, 3 and Supplementary Fig. 5-7. 68-year-old female with bilateral lung metastases and multiple mediastinal lymph node metastasis was treated with chemo-immunotherapy (carboplatin, pemetrexed, and pembrolizumab). PD-L1 expression in the pre-treatment tumor specimen was 2%. The patient had stable disease for 621 days with overall survival > 3 years.
A Contrast-enhanced cross-sectional imaging obtained at prior to and during treatment.
B Expression of CX3CR1 in peripheral blood CD8 T cells (left) and the CX3CR1 score (right) at different time points as indicated.
