# Supplementary material for: Predictive and Prognostic Implications of Circulating CX3CR1+ CD8+ T Cells in Non–Small Cell Lung Cancer Patients Treated with Chemo-Immunotherapy
Source: Cancer Res Commun. 2023 Mar 30;3(3):510–20. doi: 10.1158/2767-9764.CRC-22-0383 (PMC10060186; doi:10.1158/2767-9764.CRC-22-0383)
Supplement: Supplementary Figure S5 — Supplementary Figure 5. Related to Figure 2 and Supplementary Table 4. Analysis of peripheral blood CD45+CD19–CD3+CD56– cells by scRNA/TCR-seq. Heatmap of all cells showing the expression levels of the 10 most discriminative genes per cell type (in rows) across all the identified cell populations (in columns). Gene expression in each clusters are also listed in Supplementary Table 4. Color-code layout: scale of purple to yellow; from lowest expression to highest expression. [file crc-22-0383-s06.pdf]

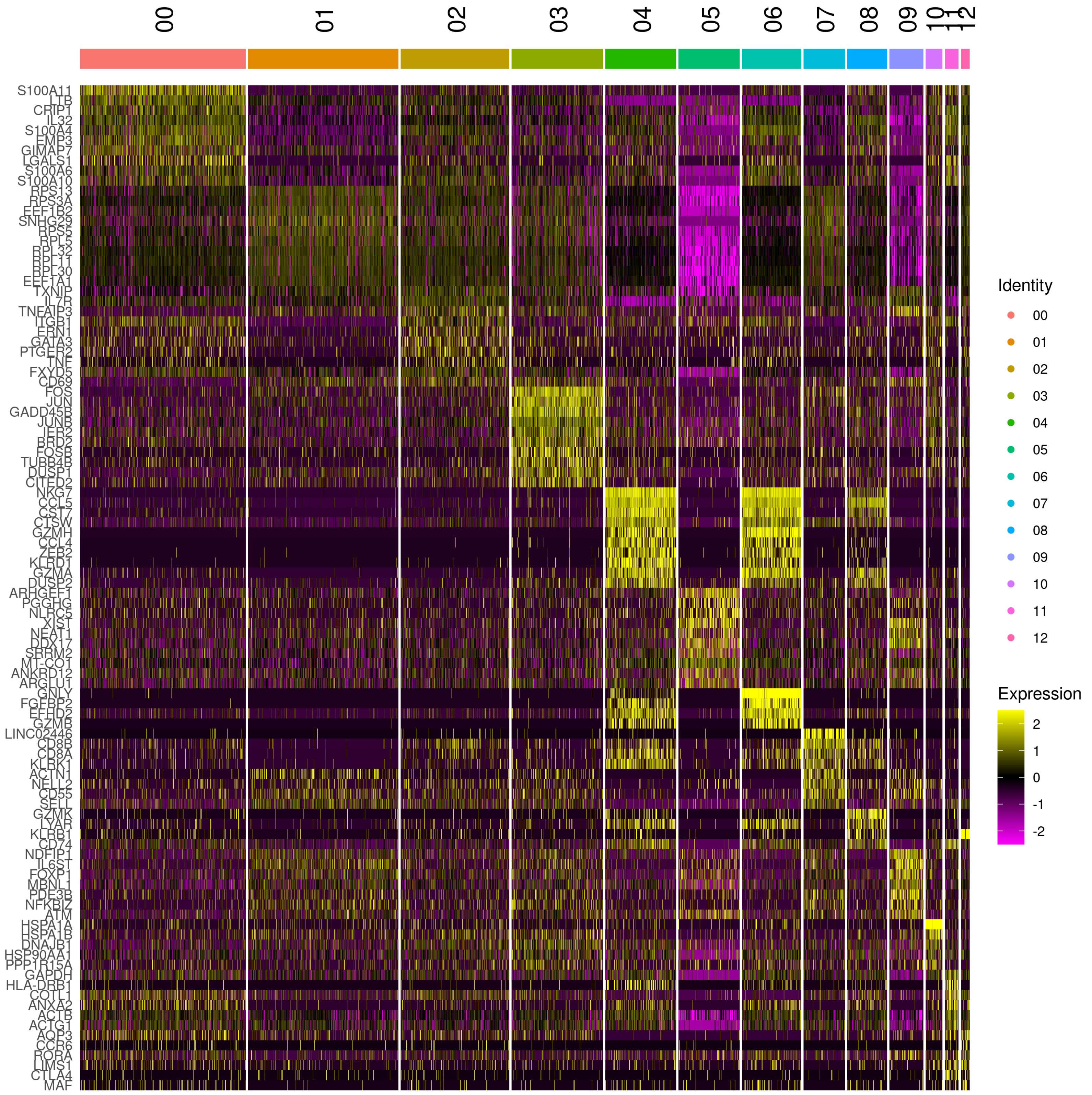

Supplementary Figure 5. Related to Figure 2 and Supplementary Table 4.
Analysis of peripheral blood CD45^{+}CD19^{-}CD3^{+}CD56^{-} cells by scRNA/TCR-seq..
Heatmap of all cells showing the expression levels of the 10 most discriminative genes per cell type (in rows) across all the identified cell populations (in columns). Gene expression in each clusters are also listed in Supplementary Table 4. Color-code layout: scale of purple to yellow; from lowest expression to highest expression.
