# Supplementary material for: Predictive and Prognostic Implications of Circulating CX3CR1+ CD8+ T Cells in Non–Small Cell Lung Cancer Patients Treated with Chemo-Immunotherapy
Source: Cancer Res Commun. 2023 Mar 30;3(3):510–20. doi: 10.1158/2767-9764.CRC-22-0383 (PMC10060186; doi:10.1158/2767-9764.CRC-22-0383)
Supplement: Supplementary Figure S6 — Supplementary Figure 6. Related to Fig. 2 and Supplementary Table 5A, B. Heat map showing the top 10 top significantly enriched pathways found in each T-cell cluster. Gene sets from Reactome (A) and Gene Ontology-Biological Processes (GO-BP) (B) are shown separately. Only gene sets with Benjamini-Hochberg-adjusted p < 0.05 were considered as significantly enriched. [file crc-22-0383-s07.pdf]

Suppl. Fig. 6

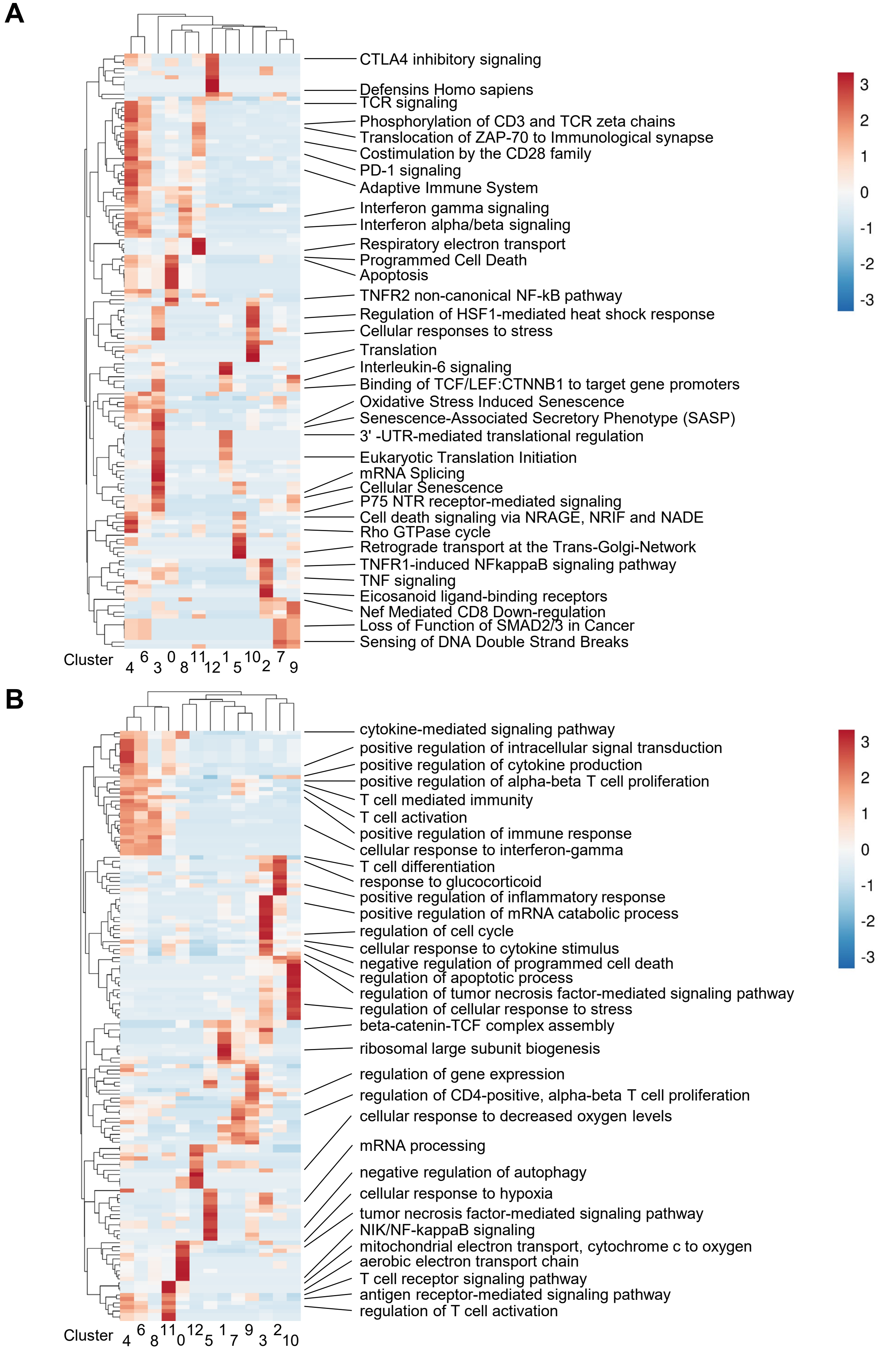

Supplementary Figure 6. Related to Fig. 2 and Supplementary Table 5A, B.
Heat map showing the top 10 top significantly enriched pathways found in each T-cell cluster. Gene sets from Reactome (**A**) and Gene Ontology-Biological Processes (GO-BP) (**B**) are shown separately. Only gene sets with Benjamini-Hochberg-adjusted $p < 0.05$ were considered as significantly enriched.
