# Supplementary material for: Predictive and Prognostic Implications of Circulating CX3CR1+ CD8+ T Cells in Non–Small Cell Lung Cancer Patients Treated with Chemo-Immunotherapy
Source: Cancer Res Commun. 2023 Mar 30;3(3):510–20. doi: 10.1158/2767-9764.CRC-22-0383 (PMC10060186; doi:10.1158/2767-9764.CRC-22-0383)
Supplement: Supplementary Figure S7 — Supplementary Figure 7. Related to Fig. 3 Bar graphs displaying the counts of tumor-infiltrating lymphocytes (TILs) expressing a given TCRβ clonotype on the y axis and individual TIL clonotypes ordered by increasing frequency on the x axis for a patient in supplementary figure 4. [file crc-22-0383-s08.pdf]

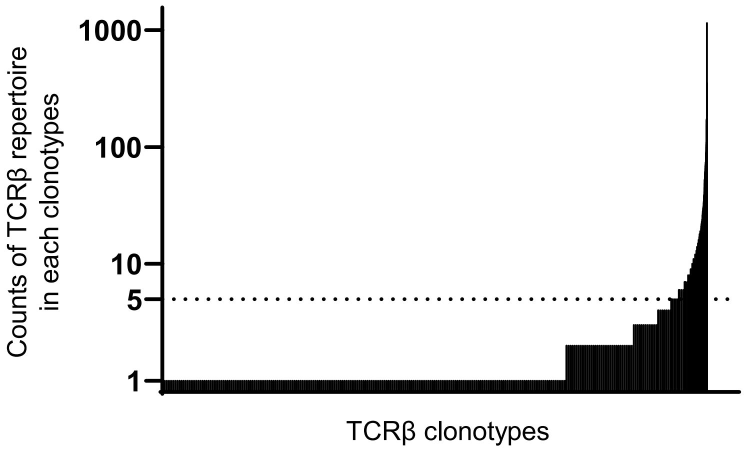

Supplementary Figure 7. Related to Fig. 3

Bar graphs displaying the counts of tumor-infiltrating lymphocytes (TILs) expressing a given TCRβ clonotype on the y axis and individual TIL clonotypes ordered by increasing frequency on the x axis for a patient in supplementary figure 4.
